# Diffusion Tensor Imaging for Diagnosing Root Avulsions in Traumatic Adult Brachial Plexus Injuries: A Proof-of-Concept Study

**DOI:** 10.3389/fsurg.2020.00019

**Published:** 2020-04-16

**Authors:** Ryckie G. Wade, Steven F. Tanner, Irvin Teh, John P. Ridgway, David Shelley, Brian Chaka, James J. Rankine, Gustav Andersson, Mikael Wiberg, Grainne Bourke

**Affiliations:** ^1^Department of Plastic and Reconstructive Surgery, Leeds Teaching Hospitals Trust, Leeds, United Kingdom; ^2^Faculty of Medicine and Health Sciences, University of Leeds, Leeds, United Kingdom; ^3^National Institute for Health Research (NIHR), Leeds Biomedical Research Centre, Leeds, United Kingdom; ^4^Department of Medical Physics and Engineering, Leeds Teaching Hospitals Trust, Leeds, United Kingdom; ^5^Leeds Institute for Cardiovascular and Metabolic Medicine, University of Leeds, Leeds, United Kingdom; ^6^The Advanced Imaging Centre, Leeds Teaching Hospitals Trust, Leeds, United Kingdom; ^7^Department of Radiology, Leeds Teaching Hospitals Trust, Leeds, United Kingdom; ^8^Department of Integrative Medical Biology (Anatomy), Faculty of Medicine, Umeå University, Umeå, Sweden; ^9^Department of Surgical and Perioperative Science (Hand and Plastic Surgery), Faculty of Medicine, Umeå University, Umeå, Sweden; ^10^Wallenberg Centre for Molecular Medicine, Umeå University, Umeå, Sweden

**Keywords:** brachial plexus (D001917), diffusion tensor imaging (D056324), spinal nerve roots (D013126), peripheral nerve injuries (D059348), neurosurgery (D009493), tractography

## Abstract

Cross-sectional MRI has modest diagnostic accuracy for diagnosing traumatic brachial plexus root avulsions. Consequently, patients either undergo major exploratory surgery or months of surveillance to determine if and what nerve reconstruction is needed. This study aimed to develop a diffusion tensor imaging (DTI) protocol at 3 Tesla to visualize normal roots and identify traumatic root avulsions of the brachial plexus. Seven healthy adults and 12 adults with known (operatively explored) unilateral traumatic brachial plexus root avulsions were scanned. DTI was acquired using a single-shot echo-planar imaging sequence at 3 Tesla. The brachial plexus was visualized by deterministic tractography. Fractional anisotropy (FA) and mean diffusivity (MD) were calculated for injured and avulsed roots in the lateral recesses of the vertebral foramen. Compared to healthy nerves roots, the FA of avulsed nerve roots was lower (mean difference 0.1 [95% CI 0.07, 0.13]; *p* < 0.001) and the MD was greater (mean difference 0.32 × 10^−3^ mm^2^/s [95% CI 0.11, 0.53]; *p* < 0.001). Deterministic tractography reconstructed both normal roots and root avulsions of the brachial plexus; the negative-predictive value for at least one root avulsion was 100% (95% CI 78, 100). Therefore, DTI might help visualize both normal and injured roots of the brachial plexus aided by tractography. The precision of this technique and how it relates to neural microstructure will be further investigated in a prospective diagnostic accuracy study of patients with acute brachial plexus injuries.

## Introduction

Approximately 1% of adults involved in major trauma sustain a brachial plexus injury (BPI) (1) which cause disability (2, 3), pain (4), psychological morbidity (5) and impaired quality of life (2, 3).

Root avulsions are the most prevalent form of injury in traumatic BPI (6). Root avulsions are high-force injuries which affect all neural elements including the anterior horn cells (7), fibers in the transitional zone and free rootlets, all of which precludes re-implantation (8) and mandates reconstruction by nerve transfer. Nerve transfers are cost-effective (9), low morbidity procedures which significantly improve function (10). Early diagnosis is of critical importance because early reconstruction improves outcomes (11, 12) and might mitigate the chronic neuropathic pain (13), which is experienced by 95% of patients with BPIs (4). Therefore, early and accurate diagnosis of root avulsion(s) is of paramount importance.

Magnetic resonance imaging (MRI) is the best non-invasive test for diagnosing traumatic brachial plexus root avulsion(s); however, its accuracy (using conventional anatomical sequences) is modest at-best and importantly, MRI misclassifies ~28% of in-continuity nerves as avulsed and fails to identify ~7% of true avulsions (6). Further, there is no consensus (nor data) on the ideal time to scan such patients or which sequences are most accurate. Therefore, there is a pressing need to improve MRI techniques to better evaluate the roots of the brachial plexus.

Diffusion tensor imaging (DTI) characterizes tissue microstructure and provides reproducible proxy measures of nerve health that are sensitive to myelination, axon diameter, fiber density, and organization (14–19). DTI is sensitive to the diffusion of water, which is anisotropic in the presence of tissue microstructure. By acquiring data that are diffusion-weighted to different degrees in multiple directions, and fitting a 3D tensor, which is analogous to a 3D ellipsoid, one is able to estimate metrics that reflect the underlying microstructure. These metrics include fractional anisotropy (FA), mean diffusivity (MD), axial diffusivity (AD) and radial diffusivity (RD). FA is a scalar value between zero and one; an FA of zero implies isotropic diffusion, whereas a FA closer to one implies diffusion that occurs preferentially along a single axis (e.g., up and down a nerve). Diffusivity parameters describe the molecular diffusion rate: the MD is the average rate, AD describes the rate of diffusion in the long axis of the tensor (e.g., up and down the nerves) and RD describes diffusion perpendicular to the long axis of the tensor (e.g., across the cross-section of the nerves). In animal models, healthy peripheral nerves have a higher FA and lower MD than injured nerves (14, 15) and DTI based tractography can identify partial and completely divided nerves 7 days after injury (20). Furthermore, DTI may be useful to surgeons in diagnosing root avulsions by examining tractograms. The literature concerning DTI of the brachial plexus is sparse, but includes healthy volunteer studies performed at 3 Tesla (21–24), injured patients studies at 1.5 Tesla (25) and neoplasms (21). There is a lack of DTI research on adults BPIs performed at 3T, and the typical measurements of diffusivity and anisotropy in these structures following injury is yet to be determined. The potential for DTI to provide a meaningful supplemental assessment of the roots (alongside current sequences) for adults with traumatic BPIs and the deficit of research on this important problem forms the rationale for this proof-of-concept study.

Our hypothesis was that at the level of a root avulsion, deterministic diffusion tensor tractography would not reconstruct tracts which represent the root. Therefore, we aimed to develop a DTI sequence to visualize the roots and compare the findings between healthy and injured patients.

## Materials and Methods

This cross-sectional study was designed and reported in accordance with the STARD guidance (26), taking into account the domains of the QUADAS-2 (27), and PRISMA-DTA (28) tools. This study was approved by the National Health Service Health Research Authority (16/YH/0162) and written informed consent was provided by all participants.

### Subjects

After a period of sequence development, DTI data from seven prospectively recruited healthy individuals (four males and three females, with a mean age of 28 years [standard deviation, SD 9] which represents the population at-risk) were acquired. Thereafter, we recruited 12 adults (all male) with unilateral brachial plexus root avulsions who were surgically explored by a single surgeon between 2009 and 2014 (with a median of 6 years [IQR 4, 7] between surgery to DTI); these patients had since been discharged from clinical services. Ten patients sustained their injuries in motorcycle collisions, one man fell from a 1st story window and one pedal-cyclist was hit by a car. The mean age at the time of injury was 30 years (SD 9) and mean age at the time of DTI was 35 (SD 10), neither of which was statistically different to the age of healthy volunteers. Individuals were excluded for standard MRI-safety concerns, claustrophobia, the inability to lie still (e.g., due to athetoid movements, dystonias, chorea, etc.), a bilateral BPI and any other neurological disorder which impaired the affected limb.

### Image Acquisition and Reconstruction

We were concerned with the ability of deterministic tractography from DTI to differentiate normal roots (no root avulsion) from abnormal roots (suspected root avulsion). DTI data were acquired at a field strength of 3 Tesla (T) using a Siemens Magnetom Prisma (Siemens Healthcare Limited, Erlangen, Germany) and single-shot echo-planar imaging (ssEPI) sequence. The acquisition parameters were as follows: 45 axial slices of 2.5 mm^3^ isotropic resolution with a field-of-view 305 × 305 × 105 mm from the C3/4 to T2/3 intervertebral discs. Twenty diffusion directions using twice refocused spin echoes were used, with 10 averages of the b0, a *b*-value of 1,000 s/mm^2^, a TrueForm B1 shim and up to 2nd order B0 shimming was performed, with the shim and imaging volumes matched to improve B0 homogeneity. An AP phase encoding direction was used with 4 repetitions averaged inline. The repetition time was 4,300 ms, echo time 66 ms, echo spacing 0.5 ms, echo train length 445 ms, GRAPPA factor 2, receiver bandwidth 2,276 Hz, interleaved with motion correction on, distortion correction off and strong fat saturation. A 64-channel head and neck coil in combination with posterior spine coils were used. The acquisition time was 6 min 41 s.

We sought to test tractography without pre-processing, using software on the operator console (Siemens NeuroLab 3D) by RGW (an Academic Plastic Surgery Registrar with 4 years of experience and formal training in diffusion tensor imaging of peripheral nerves). Seed points were manually placed to cover the cervical spinal cord in cross-section. Tracts were propagated using polylines and the following maximum thresholds: FA 0.06, 35° angle, 4 samples per voxel and 1.15 mm step lengths. Tracts were viewed by a single musculoskeletal radiologist (JJR) with 20 years of experience in brachial plexus and spinal imaging. The test was considered positive for root avulsion when there was a visible lack of continuity between the tracts in the spinal cord and the brachial plexus or an absence of a tract attaching to the spinal cord. The diagnosis of root avulsion was binary with implicit threshold. The mean (and SD) fractional anisotropy (FA) and mean diffusivity (MD) were calculated from a region of interest (ROI) placed by RGW, which consisted of five 2.5 mm^2^ pixels in the axial plane (Figure S1) covering the lateral recess of the vertebral foramen. Values for the cervical cord were derived from the corresponding cervical level.

### Reference Standard

All patients underwent surgical exploration of all roots (C5-T1) prior to recruitment. Hemilaminectomy was not performed. Somatosensory evoked potentials were not used. Avulsion was a binary outcome with implicit threshold, defined by any combination of the following: the absence of a nerve root in the exit foramina; relaxation, attenuation, and displacement of a scarred proximal nerve trunk or a visible dorsal root ganglion; no identifiable nerve fascicles on exploration of the nerve root; empty proximal nerve sheaths. If there was a neural structure in the foramen but it was easily pulled away, then avulsion was diagnosed. Other MR sequences were not used at the reference standard because these too may be inaccurate; the best possible method of determining the integrity and suitability of the root for reconstruction is direct visualization by surgical exploration.

### Statistical Analysis

Data were analyzed using Stata v15 (StataCop LLC, Texas). Age was skewed so is represented by the median and interquartile range (IQR) and compared using the Wilcoxon rank-sum test. Other scaled variables are represented by the mean (and standard deviation, SD) and compared using independent samples *t*-test. The true positive (TP), false positive (FP), true negative (TN), and false negative (FN) vales are calculated based on the findings of the index and reference tests. Significance was set at 5%.

## Results

### Diffusion Tensor Imaging: Deterministic Tractography

**Figure 1:**
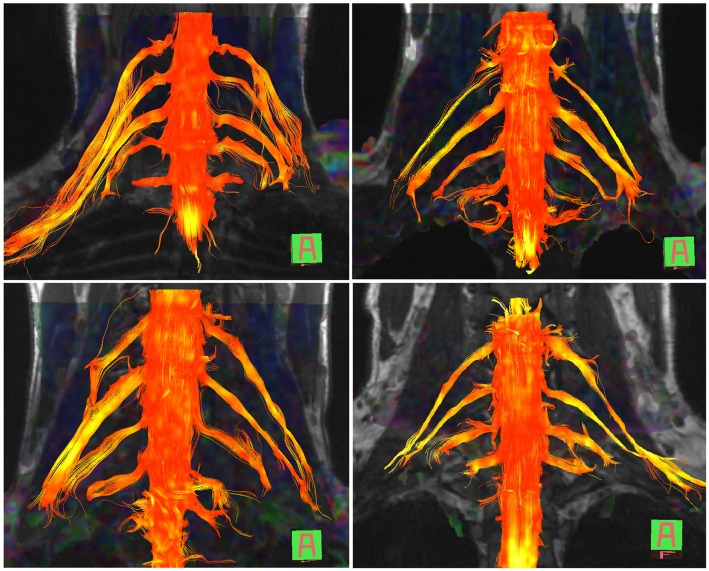
Diffusion tensor imaging tractography of the cervical cord and brachial plexus in four healthy volunteers.

**Figure 2:**
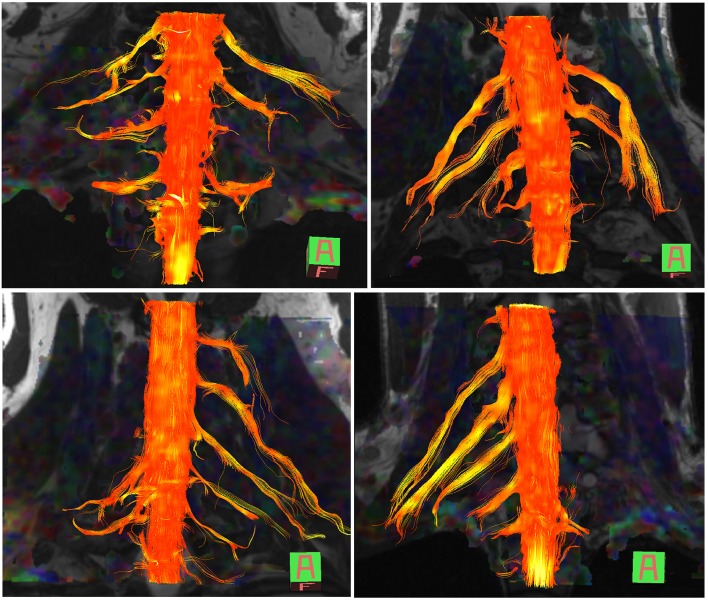
Diffusion tensor imaging tractography of the cervical cord and brachial plexus in four patients with known root avulsions. (Top left) Isolated left C7 avulsion, (top right) Left C7, C8 and T1 avulsions, (lower left) Right C5, C6 and C7 avulsions and (lower right) Left panplexus avulsion.

The normal brachial plexus is shown in four volunteers in Figure 1. Four different patterns of root avulsions are shown in Figure 2. The diagnostic accuracy of deterministic DTI for root avulsions is shown in Table 1, with an overall diagnostic accuracy of 71% (95% CI 54, 85).

**Table 1:** Diagnostic accuracy of diffusion tensor tractography (19 individuals, 190 cervical roots).

		**Diagnostic test accuracy statistics**					
		**Avulsion at operation**		**Sensitivity (95% CI)**	**Specificity (95% CI)**	**Positive predictive value (95% CI)**	**Negative predictive value (95% CI)**
		**Yes**	**No**				
Suspicion of at least one root avulsion	Yes	12	11	100 (74, 100)	58 (37, 78)	52 (31, 73)	100 (78, 100)
	No	0	15				
Absent C5 tract	Yes	3	1	50 (12, 88)	97 (84, 100)	75 (19, 100)	91 (76, 98)
	No	3	31				
Absent C6 tract	Yes	6	0	67 (30, 93)	100 (88, 100)	100 (52, 100)	91 (79, 96)
	No	3	29				
Absent C7 tract	Yes	8	1	89 (52, 100)	97 (82, 100)	89 (54, 98)	97 (81, 99)
	No	1	28				
Absent C8 tract	Yes	7	7	100 (60, 100)	77 (59, 90)	49 (34, 65)	100 (83, 100)
	No	0	24				
Absent T1 tract	Yes	5	14	100 (48, 100)	58 (39, 75)	26 (19, 34)	100 (79, 100)
	No	0	19				

Typically, the tracts representing the C5–8 roots were consistently visualized which is reflected in the high positive predictive values. However, tracts representing the T1 root were less often visualized (5/14 T1 roots in healthy controls and 7/10 T1 roots on patients' uninjured side).

Four patients had Horner's syndrome and when this was observed, the probability of an absent tract representing the T1 root was 100% (PPV 100%). However, if there were no features of Horner's syndrome, there was a 3% probability of an absent T1 root tract (NPV 97%; 95% CI 85, 100).

### Diffusion Tensor Imaging Values

**Figure 3:**
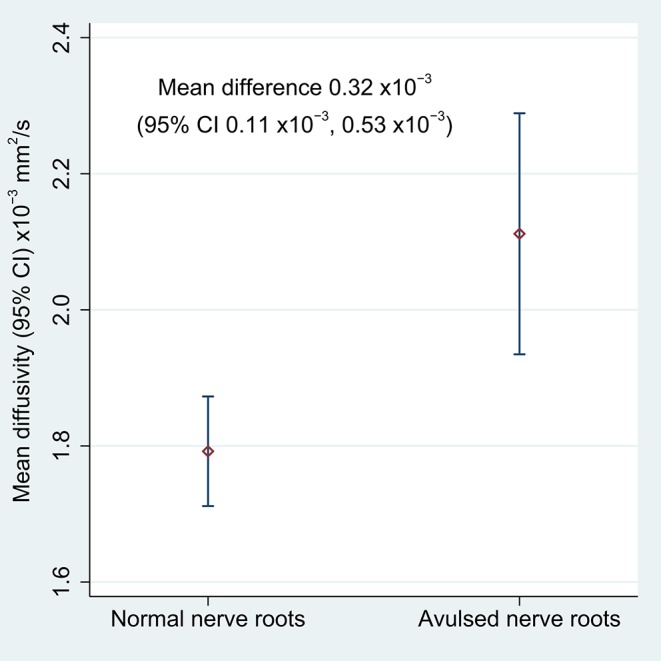
The mean diffusivity of the lateral recess of the vertebral foramen, housing either normal or avulsed C5-T1 nerve roots.

**Figure 4:**
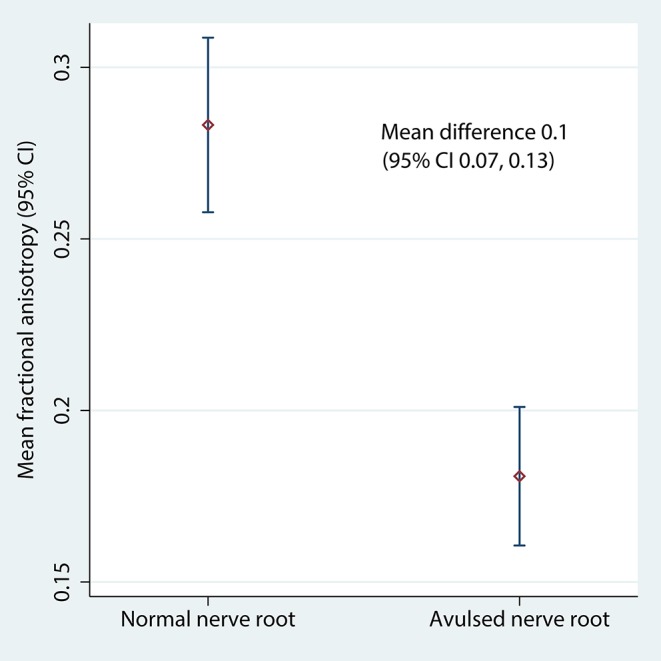
The fractional anisotropy of the lateral recess of the vertebral foramen, housing either normal or avulsed C5-T1 nerve roots.

Table 2 shows the FA and MD for the roots and corresponding levels of the cervical cord. Compared to healthy roots, the MD was 0.32 × 10^−3^ mm^2^/s higher (95% CI 0.11, 0.53; *p* < 0.001; Figure 3) and the FA 10% lower in avulsed roots (95% CI 7%, 13%; *p* < 0.001; Figure 4). The MD and FA values from the cervical cord at levels subject to avulsion injury compared to uninjured levels were not significantly different (Table 2).

**Table 2:** Diffusion measurements from the spinal cord and roots of the brachial plexus.

**Anatomical structure**	**Level**	**Mean (SD) DTI parameters**					
		**Mean diffusivity in mm**^****2****^**/s** **×** **10**^****−3****^			**Fractional anisotropy**		
		**Normal roots*^¥^***	Root avulsions^*^	***p*-value**	**Normal roots*^¥^***	Root avulsions^*^	***p*-value**
Spinal cord	C5	1.25 (0.25)	1.09 (0.23)	0.2	0.49 (0.10)	0.54 (0.09)	0.3
	C6	1.24 (0.25)	1.20 (0.21)	0.8	0.53 (0.07)	0.50 (0.07)	0.7
	C7	1.31 (0.31)	1.27 (0.22)	0.8	0.47 (0.09)	0.45 (0.08)	0.7
	C8	1.33 (0.29)	1.26 (0.21)	0.6	0.48 (0.09)	0.52 (0.09)	0.7
	T1	1.31 (0.31)	1.22 (0.24)	0.3	0.53 (0.01)	0.49 (0.10)	0.6
	Overall^∞^	1.29 (0.28)	1.21 (0.22)	0.1	0.50 (0.09)	0.51 (0.08)	0.8
Lateral recess of the vertebral foramen	C5	1.90 (0.43)	1.94 (0.33)	0.9	0.28 (0.07)	0.21 (0.08)	0.5
	C6	1.82 (0.37)	2.06 (0.40)	0.2	0.28 (0.08)	0.17 (0.05)	0.09
	C7	1.80 (0.35)	2.25 (0.39)	0.03	0.21 (0.06)	0.16 (0.03)	0.05
	C8	1.75 (0.37)	2.17 (0.27)	0.05	0.28 (0.08)	0.20 (0.05)	0.2
	T1	1.68 (0.34)	2.07 (0.43)	0.1	0.30 (0.09)	0.18 (0.05)	0.1
	Overall^∞^	1.79 (0.18)	2.11 (0.36)	0.002	0.28 (0.08)	0.18 (0.06)	0.008

* *Defined by the reference standard of operative exploration*.

¥ *In patients this is defined by the reference standard of operative exploration of the injured sides or the normal (non-explored side; all roots were defined as normal in healthy volunteers)*.

∞ *The arithmetic mean of the five levels*.

## Discussion

This study demonstrates the potential clinical utility of a diffusion tensor imaging protocol for visualizing the continuity of the roots of the brachial plexus. This technique may supplement conventional MRI sequences [which have modest accuracy at best (6)] to provide readily interpretable tractograms alongside diffusion metrics of the roots, without the need for offline pre-processing.

### Tractography in Healthy Adults

Our findings are in agreement with the limited literature concerning DTI of the brachial plexus at 3 Tesla (22–24), all of which report deterministic tractography in healthy volunteers. We based on work on that of Tagliafico et al. (22); their FA values ranged from 0.27 to 0.43 (mean 0.34) and the MD ranged from 1.4 × 10^−3^ to 1.8 × 10^−3^ mm^2^/s (mean 1.6 × 10^−3^ mm^2^/s), although they omitted to describe which anatomical structure from which these values were derived which might explain the disparity with our data. Ho et al. (23) used a 1.9 mm isotropic ssEPI sequence with 30 diffusion directions, a b-value of 800 s/mm^2^ and longer TE/TR values than us. In their report, their FA values were ~10% higher and MD 0.2 × 10^−3^ mm^2^/s lower than our data. Similarly, Oudeman et al. (24) used 3 mm isotropic EPI with 15 diffusion directions, a *b*-value of 800 s/mm^2^ and longer TE/TR values than us. Their FA and MD values were derived from the trunks and are comparable to ours (0.33 ± 0.04 vs. 0.28 ± 0.08) although again, their MD values were ~0.5 × 10^−3^ mm^2^/s lower. The differences in the MD between Ho's and Ouderman's work compared to ours and Tagliafico's might be explained by differences in the *b*-value (29) and other experimental conditions (e.g., methods of averaging, partial volume effects, etc.). Overall, our data adds to the literature and suggests that deterministic tractography and FA/MD extraction from the brachial plexus is both possible and of potential clinical utility.

### Tractography in Root Avulsions

Aside from the present work, Gasparotti et al. (25) assessed the agreement between conventional diffusion-weighted and diffusion tensor imaging for diagnosing root avulsion(s). Their offline processing corrected for artifacts and distortions caused by eddy-currents and motion, whilst we corrected for the latter inline and tested a more streamlined approach which may be preferable from a clinical perspective. Our findings suggest that universal exportation of data and pre-processing in 3rd party software may not be imperative to yield clinically meaningful tractograms of the brachial plexus. Nonetheless, more work is needed on the topics of acquisition optimisation, pre-processing and if/how these DTI-specific metrics relate to nerve microstructure.

### Diagnostic Accuracy

Specificity is arguably of paramount importance in imaging adult brachial plexus injuries (6). Gasparotti et al. (25) showed that DTI had an overall specificity of 99% and sensitivity of 85%; however, they used another form of diffusion-weighted MRI as the reference standard which is probably less accurate than surgical exploration, which is likely to inflate the estimates of accuracy. Similarly, our estimates of diagnostic accuracy may be overstated because we had knowledge of the results of the reference test (exploration).

### The T1 Root

There are a number of potential reasons to explain why we and others (22–24) are currently unable to have confidence in diffusion data acquired from the T1 root. The T1 root will be affected by susceptibility artifact due to the diamagnetic and paramagnetic effects of 1st rib and air in the apical lung, respectively, causing signal loss due to T2^*^-dephasing and mis-mapping. The proximity between the T1 root and the subclavian artery may cause flow and partial volume effects. Respiratory motion may cause mis-mapping, which cannot be fully corrected by inline or offline motion correction. Similarly, eddy-currents may cause distortion or misregistration due to spatial non-linearities and frequency/phase shifts. Overall, our data are similar to the works of Oudeman et al. (24), Tagliafico et al. (22), and Ho et al. (23). In comparison, Gasparotti et al. (25) visualized the T1 root in all cases (except three cases which were degraded by undefined artifact) which might in-part be due to the lower (1.5 Tesla) field strength and pre-processing they performed. In the future, we intend to experiment with different acquisition parameters and offline corrections for eddy-currents, motion and distortion to explore if this improves the visualization of the T1 root.

### Limitations

The diagnostic accuracy in this study is likely to be upwardly biased because we knew the pattern of avulsions and the sample was non-consecutive (non-random) (30); future work by our group is investigating the utility of preoperative DTI on a consecutive series of patients with traumatic BPIs subject to the reference standard of exploratory surgery. We imaged patients years after their injury whereas clinicians need this information is in the weeks/months after injury. DTI parameters reflect changes in the proximal and distal stumps of peripheral nerves in animals within days of injury (14, 15, 20). DTI is sensitive to Wallerian degeneration in the injured spinal cord of animals (31–34) and humans (35–38) within 3 days and for up to 1 year, respectively. DTI is also sensitive to degenerative changes in the white matter tracts of the brain over several years (39). Notwithstanding, there is a lack of research concerning DTI parameters years following peripheral nerve injury and so the effect of time can only be surmised. We believe that as the avulsed distal nerve degenerates, diffusion in the structure would regress to a similar isotropy of connective tissue (scar). Furthermore, whether DTI is useful in the acutely injured patient remains unknown and this is the subject of ongoing prospective research by our group. Moreover, there is open and ongoing debate about how diffusion-weighted images of peripheral nerves relate to the microstructure as the current technology cannot reliably differentiate the restricted diffusion of intra-axonal water from extra-cellular water elsewhere in the nerve. The ideal imaging protocol for the brachial plexus would evaluate all neural elements from the spinal cord to the target organs to detect multilevel injuries; however, this is impractical and unlikely to be achievable with finite scanning time and current technologies. Therefore, given the time-investment required for DTI, it is likely that it could currently only provide data for a specific area of the plexus, such as the roots, and should be considered as supplemental to already established protocols.

## Conclusions

This DTI sequence appears to enable the visualization of the brachial plexus without offline pre-processing, which is of potential clinical utility for diagnosing root avulsion in adults with traumatic brachial plexus injuries.

## Data Availability Statement

The raw data supporting the conclusions of this article will be made available by the authors, without undue reservation, to any qualified researcher upon reasonable written request to the corresponding author.

## Ethics Statement

The studies involving human participants were reviewed and approved by the National Research and Ethics Service of the United Kingdom (reference 16/YH/0162). The patients/participants provided their written informed consent to participate in this study.

## Author Contributions

RW, MW, and GB conceived the study. RW, GB, MW, JJR, JPR, and ST designed the study. Data were collected and MRIs performed by RW, ST, IT, JPR, DS, and BC. Aspects of image analyses were performed by RW, ST, IT, GA, JPR, and JJR. RW led the statistical analyses and prepared the manuscript. All authors edited and approved the manuscript.

### Conflict of Interest

The authors declare that the research was conducted in the absence of any commercial or financial relationships that could be construed as a potential conflict of interest.

## Supplementary Material

Figure S1 Axial MD map showing the placement of ROIs.

## Abbreviations

BPI: brachial plexus injury
DTI: diffusion tensor imaging
FA: Fractional Anisotropy
MD: Mean Diffusivity
MRI: magnetic resonance imaging
ROI: region of interest
ssEPI: single shot echo planar imaging.
