# Supplementary figures and images for: Diffusion Tensor Imaging for Diagnosing Root Avulsions in Traumatic Adult Brachial Plexus Injuries: A Proof-of-Concept Study
Source: Front Surg. 2020 Apr 16;7:19. doi: 10.3389/fsurg.2020.00019 (PMC7177010; doi:10.3389/fsurg.2020.00019)

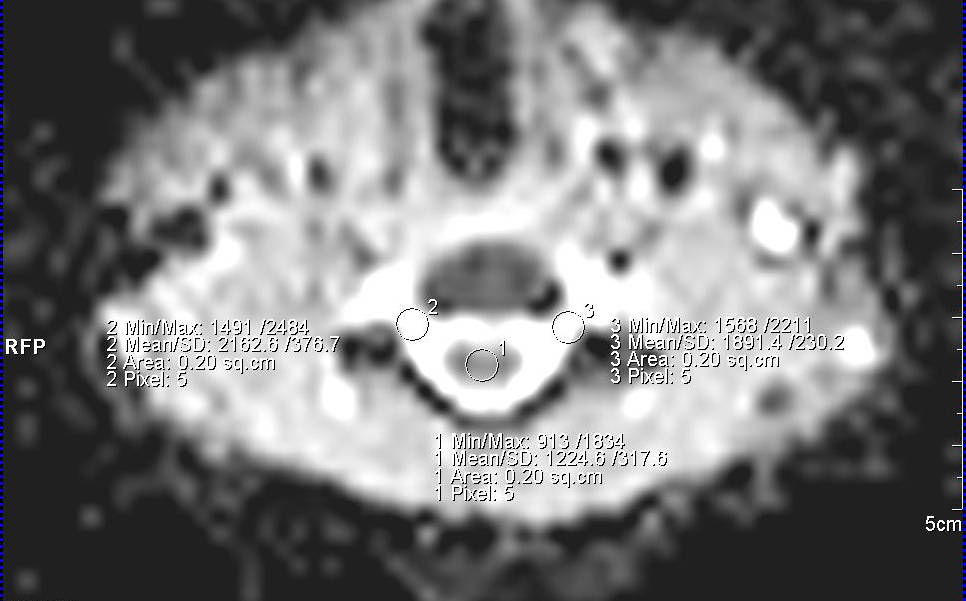

Supplement: Figure S1 — Axial MD map showing the placement of ROIs. [file Image_1.JPEG]
